# PSMA Theranostics in Prostate Cancer and Beyond: Current and Future Perspectives

**DOI:** 10.3390/cancers17223717

**Published:** 2025-11-20

**Authors:** Kieran Sandhu, David Chen, David Hennes, Declan G. Murphy, Nathan Lawrentschuk, Marlon Perera

**Affiliations:** 1 Division of Cancer Surgery, Peter MacCallum Cancer Centre, Melbourne, VIC 3000, Australia; 2 University of Cambridge, Cambridge CB2 1TN, UK; 3 Department of Surgery, University of Melbourne, Melbourne, VIC 3010, Australia; 4 Department of Urology, Royal Melbourne Hospital, Melbourne, VIC 3050, Australia; 5 EJ Whitten Prostate Cancer Research Centre, Epworth Healthcare, Melbourne, VIC 3002, Australia; 6 Department of Urology, Austin Hospital, Heidelberg, VIC 3084, Australia

**Keywords:** prostate cancer, PSMA, PSMA-PET/CT, [^177^Lu]Lu-PSMA-617, theranostics, precision oncology

## Abstract

Prostate-specific membrane antigen (PSMA) is a type II transmembrane glycoprotein that has become central to prostate cancer (PCa) diagnostics and treatment. Beyond its enzymatic role in folate and glutamate metabolism, PSMA is upregulated in advanced PCa, where it contributes to angiogenesis, tumour progression, and therapeutic resistance. This review integrates current understanding of PSMA biology with an emphasis on the role of PSMA expression and the hallmarks of cancer-proliferative signalling, metabolic adaptation, and evasion of cell death. While PSMA has revolutionised theranostic strategies in PCa, its utility as a sole biomarker is limited in select cases such as neuroendocrine differentiation and discordant disease biology. To address these challenges, we highlight emerging biomarkers and novel imaging markers that complement PSMA, including genomic alterations, circulating tumour markers, and exosomal microRNAs. Advances in radiomics and dual-tracer positron emission tomography (PET) further refine patient selection by capturing aggressive low-PSMA phenotypes. Furthermore, PSMA-PET is showing promise in other malignancies, including renal cell carcinoma (RCC) and glioblastoma multiforme (GBM), where neovasculature expression may extend its theranostic applications beyond PCa. By situating PSMA within this broader biomarker landscape, we outline opportunities for theranostic integration, including predictive models, combination therapies and expansion into non-prostate malignancies. Understanding the biology of PSMA in conjunction with novel biomarkers provides a framework for optimising theranostic applications and advancing personalised cancer care.

## Simple Summary

Prostate-specific membrane antigen (PSMA) has changed the diagnosis and treatment of prostate cancer through the development of highly targeted imaging and radioligand therapy. This review summarises the normal function of PSMA and its role in cancer. We highlight the limitations of PSMA as a sole biomarker and discuss emerging genomic, circulating, and imaging biomarkers that can complement PSMA-based theranostics. The scope of PSMA use is being expanded by quantitative imaging, artificial intelligence, and liquid biopsies which enable prompt assessment of disease biology and treatment response. Furthermore, PSMA expression on blood vessels extends its potential beyond prostate cancer into other malignancies. Collectively, these highlight the role of PSMA in an evolving, biomarker-driven approach to personalised and precision oncology.

## 1. Introduction

The emergence of molecular imaging and targeted therapy—theranostics—has transformed, and expanded, the field of precision oncology. Prostate-specific membrane antigen (PSMA) is central to this, enabling sensitive disease localisation with PSMA-positron emission tomography/computed tomography (PSMA-PET/CT) and lesion-directed radioligand therapy (RLT) using α-β-emitting agents for patients with prostate cancer (PCa) [1,2]. Randomised trials including TheraP and VISION have validated these approaches [2,3]. Nonetheless, PSMA biology, imaging interpretation, and therapeutic applications remain dynamic with not all lesions and patients responding equally. The future of precision oncology will depend on the integration of complementary biomarkers to refine patient selection, predict therapy response, and overcome resistant disease. This narrative review synthesises the current understanding of PSMA biology and its theranostic translation, incorporating recent developments in quantitative imaging, biomarkers, emerging molecular correlates, and exploring the role of PSMA beyond PCa.

## 2. PSMA Biology

### 2.1. Genetics and Structural Profile

PSMA is encoded by folate hydrolase 1 (*FOLH1*) on chromosome 11p11.2, producing a 750 amino acid type II transmembrane glycoprotein functioning as a zinc metalloenzyme [4,5,6]. PSMA exists as a homodimer, with each monomer containing a central binding cavity with two zinc ions and entrance accommodating glutamate moieties [7]. PSMA catalyses the hydrolysis of polyglutamated folates which regulates folate and glutamate metabolism [8]. These catalytic properties are important components of metabolic pathways often upregulated in malignancy [9]. In the context of RLT, ligand binding triggers clathrin-mediated endocytosis facilitating delivery of radio-labelled payloads and concentration within cancer cells [10]. Cryoelectron microscopy and crystallographic analyses have refined understanding of PSMA’s binding pocket, with the identification of key residues that interact with the glutamate-containing motifs present in most RLT [11]. These structural insights guide the rational design of next-generation ligands with improved tumour retention and reduced off-target salivary gland and marrow uptake.

### 2.2. Regulation, Expression, and Functional Roles

PSMA expression is tightly regulated by androgen-receptor (AR) signalling, chromatin accessibility, and methylation status of chromosome 11p [12]. Along the PCa disease continuum, there is dynamic modulation with upregulation occurring during the transition from hormone-sensitive to castration-resistant states, and increased expression following AR-pathway inhibitor (ARPI) administration [13]. Androgen deprivation induces PSMA transcriptional upregulation whilst neuroendocrine differentiation can suppress PSMA expression, as neuroendocrine cells are largely devoid of PSMA [12]. Both inter- and intra-lesional, spatial, and temporal heterogeneity can complicate interpretation and therapy planning.

PSMA expression is modulated at a chromatin level. Looping between enhancing and promoter regions, differential methylation, and histone-modification patterns can either activate or silence *FOLH1* transcription, explaining why some high-grade tumours are PSMA-negative despite aggressive histology [14]. DNA-methylation loss at regulatory *CpG* (5′-C-phosphate-G-3′) foci and histone-acetylation-gain polymorphisms have been linked to PSMA-high phenotypes, whereas neuroendocrine differentiation is associated with promoter hypermethylation, chromatin compaction, and reduced PSMA expression [14,15]. PSMA expression may also be influenced by microenvironmental factors including hypoxia, oxidative stress, and cytokine signalling, which may upregulate angiogenic factors and PSMA, including Hypoxia-inducible factor-1 (HIF-1α) [14,16,17].

### 2.3. Functional Roles

PSMA contributes to tumour progression through metabolic remodelling and angiogenesis, enhancing tumour cell invasion and migration via activation of focal-adhesion kinase and integrin-signalling pathways [4]. Recent reports have demonstrated that PSMA is expressed not only in PCa epithelium but also in the endothelial cells of tumour neovasculature across multiple malignancies supporting its exploitation as a vascular theranostic target [18,19,20]. The dual localisation of PSMA in both the epithelial tumour cell and surrounding neovasculature suggests a dual biologic function—sustaining the cancer metabolism and remodelling the vascular niche to sustain increased metabolic activity.

## 3. Clinical Role of PSMA

### 3.1. Imaging Biomarkers

PSMA-PET/CT has outperformed conventional imaging for both initial staging and biochemical-recurrence (BCR) localisation, with multiple trials supporting its superiority [1,2]. The proPSMA trial established that PSMA-PET/CT has a 27% higher diagnostic accuracy than combined CT and bone scan for primary staging, identifying additional nodal and distant metastases in approximately 33% of patients [1]. Meta-analyses have confirmed that standardised uptake volume (SUV)_max_ and total PSMA-positive tumour volume correlate with Gleason grade, prostate-specific antigen (PSA) levels, and risk of BCR [21,22]. The utility of SUV_max_ was explored with the recent post hoc analysis and development of a PRIMARY score, aiding diagnosis of PCa [23].

PSMA^−^ states occur in approximately 3–5% of intermediate-to-high-risk cases despite aggressive histology, highlighting biological divergence and heterogeneity [24]. Nonetheless, quantitative PSMA metrics now serve as imaging biomarkers guiding management—baseline uptake predicts outcomes of ARPI administration , chemotherapy, and RLT [25,26,27].

PSMA-PET/CT demonstrates dynamic regulation. Uptake tends to increase with disease progression—from localised hormone-sensitive states (HSPC) to metastatic castration-resistance PCa (mCRPC) [1,12,15,28]. This reflects both cellular upregulation of PSMA and clonal selection under androgen deprivation across the PCa continuum [12]. ARPIs, such as Enzalutamide, transiently augment PSMA expression and enhance imaging contrast, while neuroendocrine differentiation suppresses uptake and poses challenges for therapeutic targeting [29,30,31,32].

Increasingly, PSMA-PET/CT is integrated into radiotherapy planning and response monitoring. Radiotherapy guided by biology utilises PET signal intensity to tailor dose planning, while changes in uptake post-therapy provide response surrogates which can provide increasingly personalised care [33,34].

### 3.2. Radioligand Therapy

Following TheraP and subsequently the VISION trials, [^177^Lu]Lu-PSMA-617 is an option for patients with highly PSMA-avid mCRPC [3]. The phase II TheraP trial confirmed that [^177^Lu]Lu-PSMA-617 had a higher PSA ≥ 50% response rates (66% vs. 37%) and fewer grade-3 adverse events compared to Cabazitaxel, but no overall-survival difference [3]. The phase III VISION trial demonstrated that [^177^Lu]Lu-PSMA-617 significantly improved overall survival (median 15.3 vs. 11.3 months) and radiological progression-free survival (8.7 vs. 3.4 months) versus standard of care [2]. Although the incidence of adverse events was higher in the treatment arm (52.7% vs. 38.0%), quality of life was not impacted [2]. α-emitters strategies including ^225^Ac-PSMA-617 and ^212^Pb-PSMA are being explored to overcome resistance in those patients with bulky disease states, but validation in larger studies is necessary [35,36,37,38].

Combination regimes are emerging—PSMA-RLT with poly ADP ribose polymerase (PARP) inhibition (LuPARP trial), Docetaxel (UpFrontPSMA) are being explored [39,40]. The interim results from the first dual-tracer RLT trial (AlphaBet) were recently published demonstrating a reduction in PSA of at least 50% in approximately 50% of patients, with grade-3 events only reported in five patients [41]. To explore the synergistic benefit of [^177^Lu]Lu-PSMA-617 and surgery, men with high-risk localised PCa were given upfront [^177^Lu]Lu-PSMA-617 prior to radical prostatectomy in the LuTectomy study, demonstrating low rates of BCR and low rates of adverse events on follow-up [21,42].

Quantitative imaging metrics inform therapy eligibility and response prediction. Baseline SUV_max_ correlates with both PSA response and survival following RLT, with patients with extensive PSMA^−^ disease demonstrating attenuated benefit to RLT, underscoring the importance of multimodal biomarker assessment [43,44].

Monitoring of therapy and dynamic adaptation based on patient response is an emerging space. Serial PSMA-PET/CT assessment facilitates dynamic evaluation of treatment efficacy, with new PSMA^−^ lesions suggesting clonal escape. To manage these complexities, adaptive dosage algorithms, adjusting cumulative [^177^Lu]Lu-PSMA-617 activity based on residual uptake, are being examined to individualise treatment cycles [43,45,46]. Recently, standardised response frameworks such as Response Evaluation Criteria in PSMA Imaging (RECIP) have been proposed to integrate software-based quantitative assessment of PSMA^+^ total tumour volume [47]. Gaftia et al. found excellent agreement between visual and quantitative RECIP, further demonstrating that RECIP progressive disease was associated with significantly shorter overall survival compared with non-progressive disease [48]. However, these adaptive strategies warrant validation in larger, prospective trials. Overall, RLT toxicity profiles remain favourable—xerostomia and fatigue are the most common, while haematological toxicity remains uncommon and typically reversible. Protective strategies such as salivary-gland cooling are under active investigation [49]. Overall, RLT toxicity profiles remain favourable—xerostomia and fatigue are the most common, while haematological toxicity remains uncommon, and typically reversible. Protective strategies such as salivary-gland cooling are under active investigation and require validation in larger studies [49].

### 3.3. Determinants of Response

**Figure 1:**
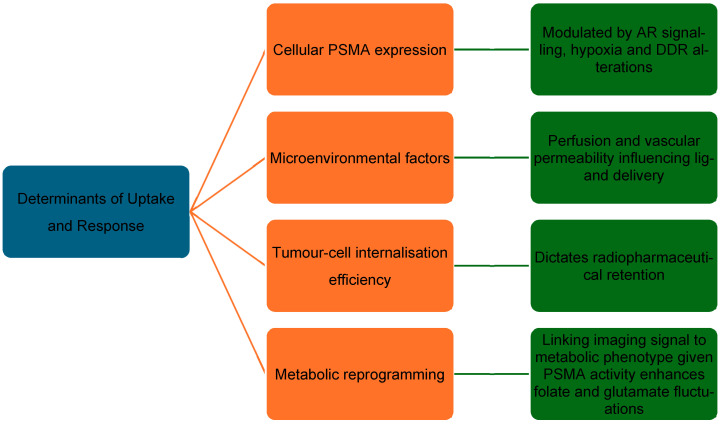
Determinants of PSMA uptake and response. DDR: DNA damage response.

PSMA uptake depends on receptor density, perfusion, cellular internalisation kinetics, and the tumour microenvironment (TME) (Figure 1) [12]. Recent meta-analyses suggest that SUV_max_ independently predicts progression-free and overall survival post-RLT in the mCRPC setting [26,43,50]. However, discordant FDG^+^/PSMA^−^ lesions signify dedifferentiation and poor response, warranting dual-tracer imaging [51,52,53].

## 4. Emerging Biomarkers to Complement PSMA

### 4.1. Limitations of PSMA as a Sole Biomarker

Despite its clinical success, PSMA is an imperfect universal marker. Heterogeneity of expression, neuroendocrine differentiation/transformation, and transcriptional suppression through epigenetic reprogramming can lead to false-negative imaging [12,13]. Off-target uptake in salivary glands and kidneys limits contrast uptake and may result in adverse effects. Small-volume disease may evade detection due to partial-volume effects and may be mitigated by artificial intelligence (AI) and machine learning (ML)—particularly deep learning-based reconstruction and partial-volume correction networks which work to enhance spatial resolution and quantitative accuracy [54].

### 4.2. Genomic and Molecular Markers

Defects in DNA damage response (DDR) genes including breast cancer type 1/2 susceptibility gene (*BRCA1/2*)*,* ataxia-telangiectasia mutated (*ATM*), checkpoint kinase 2 (*CHEK2*)*,* and partner and localiser of BRCA2 (*PLAB2*) are common in advanced disease, occurring in approximately 12% of metastatic PCa cases, and may upregulate PSMA expression via replication stress and metabolic demand [55,56]. These alterations underpin combination strategies pairing RLT with PARP inhibition and are currently being investigated in large trials such as LuPARP [39]. Phosphatase and tension homolog (*PTEN*) loss activates downstream growth signalling. Dual inhibition of PSMA and growth signalling stimulated by *PTEN* loss represent a promising therapeutic opportunity, exploiting PSMA-induced nutrient uptake while blocking downstream growth pathways [53,57,58]. The *AR-V7* splice variant confers resistance to ARPIs and identification of *AR-V7*-positive patients can prioritise PSMA-RLT or combination approaches.

Further subtype-specific PSMA biology has been elucidated with transcriptional profiling. Erythroblastosis virus E26 (*ETS*)-fusion-positive tumours, such as transmembrane-protease and serine-2-ETS-related gene (*TMPRSS2-ERG*), demonstrate stronger *FOLH1* promote activity [12,56]. Integration of these molecular subtype findings with PSMA imaging signatures could help refine patient stratification for RLT [59]. Epigenetic modifications are now emerging as biomarkers. Histone-deacetylase inhibitors and demethylating agents can re-induce *FOLH1* expression in low-expression PSMA tumours, potentially making them susceptible to PSMA-targeted radioligands, but this remains validated only in vitro and requires further exploration [14].

### 4.3. Circulating Biomarkers

Liquid biopsies provide longitudinal insight. Circulating tumour DNA (ctDNA) mirrors mutational burden and clonal evolution, while circulating tumour cell (CTC) enumeration and phenotyping can track tumoral heterogeneity [60,61]. ctDNA profiling can help detect DDR and *PTEN* alterations that predict PSMA-RLT responsiveness, and serial ctDNA sampling may provide insight into emergent mutations that may signal developing RLT resistance. Quantification of total ctDNA may serve as a biomarker of advanced PCa [62,63]. Phenotyping and CTC enumeration provide some prognostic value—high baseline CTC counts correlate with shorter progression-free survival, and detection of PSMA^+^ CTCs can complement imaging in the assessment of intra-lesional heterogeneity [64]. Therapeutic response can be predicted by measuring exosomal miRNA such as miR-141 as well as mRNA cargo reflecting *FOLH1,* DDR, or *AR-V7* status [65]. The integration of exosomal and PSMA-PET/CT data could enable real-time adaptive therapeutic approaches—escalating RLT with miRNA spikes or de-escalating when ctDNA clears.

### 4.4. Imaging Biomarkers

Radiomics derived from PSMA-PET/CT, including texture, entropy, and total lesion volume, correlate strongly with outcomes beyond SUV_max_ [13,66]. There exist multiple other targets that may complement the use of PSMA-tracer imaging in challenging circumstances of low-uptake disease states (Table 1). These alternative molecular targets illustrate a shift toward multi-target theranostics, replacing single-antigen approaches to address tumour heterogeneity which is common in advanced disease states. While these radiomics show promise, implementation into routine clinical decision-making faces challenges due to a lack of cross-centre standardisation, small cohorts, and variations in reconstruction protocols across centres. Thus, there is need for multi-institutional radiomic repositories and data harmonisation frameworks to resolve these limitations [67]. Furthermore, data privacy and interoperability are essential for any deployment of large-scale AI models.

### 4.5. Tumour Microenvironment Markers

PSMA expression interacts with the TME and can influence theranostic response. Hypoxia reduces β-particle effectiveness and may induce PSMA downregulation through metabolic reprogramming and vascular endothelial growth factor (*VEGF*)-driven angiogenesis [53,78,79]. To address these limitations, hypoxia-PET tracers, such as ^18^F-FAZA and ^64^Cu-ATSM, are used to guide patient selection and RLT [80,81]. Stromal and angiogeneic factors—*VEGF* and HIF-1α—correlate with PSMA uptake on endothelial cells [16,82]. Given that PSMA is expressed on the endothelial cells of tumour neovasculature, these angiogeneic pathways may be exploited to amplify and concentrate ligand delivery, explaining the strong uptake in highly vascular tumours such as renal cell carcinoma (RCC) and glioblastoma multiforme (GBM). RLT has a complex interplay with the tumour microenvironment, with a recent review by Eapen et al. suggesting that beyond PSMA^+^-cell death, [^177^Lu]Lu-PSMA-617 treatment may stimulate a systemic immune response which may be harnessed to produce long-lasting cancer immunity [17].

### 4.6. Immune Markers

Immune checkpoint biomarkers represent another relevant pathway of exploration. High PSMA expression co-exists with increased programmed death ligand 1 (*PD-L1*) expression in subsets of men with PCa, supporting the need for combined RLT and immunotherapy strategies [83,84]. There are now early-phase trials, such as PRINCE, that have shown tolerability and potential additive efficacy of RLT and Pembrolizumab [85].

The future of PSMA-based theranostics, and more broadly RLT depends on a multimodal approach. Combining genomic factors (DDR, *PTEN*, *AR-V7* variants, PSMA mRNA), radiomic factors (SUV_max_-based metrics), and liquid biopsy data (ctDNA/CTCs/exosome counts) can generate composite risk models to guide individualised treatment algorithms. AI may augment these approaches and provide robust means to predict RLT response and toxicity with high accuracy. These frameworks will move PSMA theranostics into a new era of dynamic, personalised precision medicine.

## 5. PSMA Theranostics Beyond PCa

PSMA is expressed on the endothelium of tumour neovasculature in non-PCa malignancies, reflecting the role of the enzyme in angiogenic states and highlighting its utility beyond the prostate. This provides promise for enabling vascular-targeted imaging in a multitude of tumours.

### 5.1. Renal Cell Carcinoma (RCC)

PSMA is highly expressed in the endothelial cells of clear-cell RCC neovasculature [86]. In a recent systematic review by Sadaghiani et al., PSMA-PET/CT demonstrated promise in the restaging setting, but the review was limited by small patient numbers and heterogeneity necessitating further research [87]. In several prospective series, ^68^Ga-PSMA-11 and ^18^F-DCFPyL PET identified nodal and pulmonary metastases missed by conventional imaging, with uptake intensity correlating with histologic grade [88,89]. Prospective trials are now under way to examine [^177^Lu]Lu-PSMA RLT in metastatic RCC [90,91].

The role of PSMA-PET/CT may extend beyond detection of occult disease in RCC. In their study, Khaleel et al. assessed the correlation between *FOLH1* expression and gene expression signature scores that corresponded to the TME [91]. Increased *FOLH1* expression was significantly associated with higher TME angiogenesis, and expression predicted progression-free survival in patients with metastatic clear-cell RCC treated with the tyrosine kinase inhibitor Sunitinib [91]. This suggests that PSMA-PET/CT imaging could be utilised as a non-invasive marker to guide systemic therapy options and predict the treatment response to VEGF-inhibiting agents in patients with metastatic clear-cell RCC.

### 5.2. Salivary-Gland Tumours

Adenoid cystic carcinoma (ACC) of the salivary glands often exhibits strong PSMA uptake in tumour cells and neovasculature [92]. ^68^Ga-PSMA PET identifies primary and metastatic ACC lesions with high contrast [92]. Although a small pilot study, Wang et al. found that amongst patients diagnosed with ACC, ^68^Ga-PSMA PET revealed more PET^+^ extrapulmonary tumours than ^18^F-FDG PET, but fewer PET^+^ pulmonary lesions, suggesting that a combination of both imaging modalities may be optimal in patients with ACC [93]. Nonetheless, prospective multi-centre studies are necessary to validate these findings. Prospective case series have reported mixed efficacy profiles in patients treated with [^177^Lu]Lu-PSMA therapy for ACC, but treatment has been well tolerated with rapid relief of tumour-associated discomfort [94,95].

### 5.3. Glioblastoma Multiforme (GBM)

GBMs express PSMA in tumour microvessels and in reactive astrocytes [82]. Immunohistochemistry studies have demonstrated PSMA staining in GBMs, but this has not correlated with tracer uptake [96]. ^68^Ga-PSMA PET detects regions of active glioma with higher specificity than conventional imaging [97]. Given that PSMA expression in endothelial-targeted PSMA-RLT acts as vascular-targeted radiotherapeutic agent. Earlier clinical experience with [^177^Lu]Lu-PSMA in recurrent GBM has demonstrated feasibility and safety, with stabilisation of disease in pre-treated patients, but requires validation in larger series [98,99].

### 5.4. Translational Insights

Across non-PCa malignancies, PSMA expression tends to be localised to proliferating endothelial cells within the architecture of neovasculature, rather than the malignant epithelial pool. This expands the functioning definition of PSMA from a prostate-specific antigen to a pan-angiogenic marker of tumour microvasculature. These findings provide promising insights for the future of oncological therapy. PSMA ligands may act as vascular-targeted radiotherapeutics, providing selective ablation of tumour blood supply.

### 5.5. Clinical Challenges

Despite promising early results, several challenges remain: (1) Heterogenous PSMA expression across tumour types even within a single lesion necessitates companion imaging to accurately select patients. (2) Physiological uptake in off-target organs complicates imaging interpretation and dosimetry. (3) Randomised trials are lacking with most data derived from small single-centre cohorts with an undefined state for appropriate dosimetry in non-prostate malignancies. (4) Ligand optimisation is needed to increase vascular endothelial penetration and limit off-target binding. Nevertheless, a new theme of PSMA marking pathological angiogenesis has emerged, positioning it as a promising target for vascular theranostics. Further studies may build on these findings by utilising a multi-tracer approach to capture both the tumour and surrounding neovasculature, providing a holistic map of the tumour, perfusion, and metabolism.

## 6. Future Directions

A future direction in PSMA theranostics is the integration of geonomic, imaging, and circulating biomarkers into multimodal predictive frameworks. These models should include a combination of PSMA-PET metrics with ctDNA, DDR gene status, and transcriptomic signatures to guide appropiate patient selection for therapy [63,100].

### 6.1. Quantitative and AI-Driven Imaging

The evolution of PSMA theranostics is being driven by ongoing exploration of quantitative imaging metrics including SUV_max_, PSMA-derived tumour volume, and total lesion PSMA uptake [13]. These serve as imaging biomarkers to predict and evaluate therapy response and may be combined with genomic features to form a hybrid framework linking PSMA-PET/CT phenotypes with genomic features. AI and ML models are transforming PSMA-PET interpretation from solely qualitative to predictive analytical tools [101].

### 6.2. Adaptive and Combination Therapy

Traditionally, PSMA-RLT protocols have used fixed cycles, but adaptive dosing guided by imaging and biomarkers is a promising approach on the horizon. Serial PSMA-PET and liquid biopsy may enable real-time therapy adaptation, with rising ctDNA or the appearance of PSMA- lesions triggering escalation to α-emitter therapy, or a switch to combination regimes. On the other hand, biochemical and imaging responses could justify treatment de-escalation, minimise toxicity, and reduce costs. The future of PSMA-based therapy is combinatorial, integrating molecularly matched agents to overcome resistant clones. Sequential α/β-emitter regimes are gaining traction [41]. Recently, Kluge et al. examined the relationship between cell-free DNA levels and PSMA-positive tumour volume, finding a weak correlation [62]. This is contrary to Amseian et al., who found no correlation in their prospective study, emphasising the need for larger, prospective studies [102].

### 6.3. Next-Generation Ligands and Isotopes

Ligand engineering aims to optimise tumour retention and clearance kinetics, minimising off-target internalisation. Modifications to linker length, charge, and hydrophilicity have yielded ^64^Cu-PSMA-CM ligands with improved albumin binding and extended circulation time and more are in development to improve binding to PSMA and minimise off-target toxicity [103]. Bispecific ligands combining *GRPR* or *FAP* motifs are being validated, aiming to overcome inter-lesional heterogeneity by binding multiple antigens simultaneously, ensuring radioligand delivery even when a single antigenic target is downregulated [68,104]

### 6.4. Future Outlooks

The next horizon is the integration of imaging, molecular, and clinical data in predictive models. In their models, Gafita et al. developed a normogram that combined PSMA-PET/CT-derived tumour volume, SUV_mean_ and baseline clinical features to predict overall survival following [^177^Lu]Lu-PSMA-617 therapy [43]. Similarly, Pan et al. used a dual-tracer approach to identify PSMA^−^/FDG^+^ discordant disease in a multi-centre study to develop nomograms and predict poor RLT response [105].

Despite these initial results, validation is necessary to ensure cross-centre reproducibility. Standardisation of reconstructive protocols, such as European Association of Nuclear Medicine (EANM)/EANM Research Ltd. (EARL)-compliant imaging, is essential to ensure consistentcy across institutions [106]. Given significant concern surrounding privacy, federated-learning approaches enable collaborative model training without direct data sharing [107]. Adherence to reporting standards such as the Transparent Reporting of a multivariable prediction model for Individual Prognosis Or Diagnosis + AI (TRIPOD+AI) will improve transparency of studies developing prediction models [108]. Collectively, these strategies support the development of reproducible predictive models that can guide adaptive PSMA theranostics.

The convergence of imaging, therapy, and AI marks the beginning of a new era in theranostics, with PSMA remaining at the cornerstone of this transformation, defining the next generation of precision oncology. In the next decade, PSMA-directed RLT may evolve from a salvage option for mCRPC to an earlier-line, combination-based, cross-tumour modality. Nonetheless, it is important that these resources are distributed equally across healthcare services as they emerge to avoid inequalities [109,110].

## 7. Conclusions

PSMA has ushered in a new era of successful, targeted, molecular theranostics, but PSMA expression is dynamic and context-dependent. Quantitative imaging metrics, genomic signatures, and microenvironmental cues can refine patient selection and guide combination approaches. The future or precision oncology will integrate PSMA with a constellation of biomarkers to personalise theranostic strategies. Moreover, PSMA’s endothelial expression across multiple malignancies positions it as the gateway to a new era of biomarker-driven pan-cancer theranostic strategy. Nonetheless, future research is needed to standardise imaging and molecular criteria to define PSMA- disease, as well as integration of multi-omic signature into decision algorithms and prospective trials to confirm their utility in appropriate patient selection and adaptive dosing regimens.

## Figures and Tables

**Table 1:** Biomarkers under investigation for targeting.

Biomarker	Function/Utility
Gastric-releasing peptide receptor (*GRPR*) [68,69,70]	• Complementarily expressed to PSMA, particularly in earlier-stage or low-grade PCa; • ^68^Ga-RM2 and ^64^Cu-CBRM have demonstrated high tumour-to-background ratios in PSMA-negative lesions; • Early-phase clinical evaluation of GRPR-targeted radioligands and antagonists (e.g., [^177^Lu]Lu-NeoBOMB1, [^177^Lu]Lu-RM2).
Fibroblast activation protein (*FAP*) [71,72,73]	• Highly expressed on cancer-associated fibroblasts within tumour stroma; • Represents pan-tumour target; • FAPI-PET provides rapid kinetics, and exceptional lesion contrast; • Strong uptake in PSMA-negative or dedifferentiated lesions; • [^177^Lu]Lu-FAPI radioligand therapies have entered first-in-human pilot studies.
Six-transmembrane epithelial antigen of prostate (*STEAP1*) [74,75]	• Cell-surface metalloreductase overexpressed in up to 90% of prostate tumours, independent of PSMA status; • Expression of *STEAP1* persists in PSMA-low and neuroendocrine-like variants; • Early-phase clinical trials with bispecific antibody Xaluritamig.
Urokinase-type plasminogen-activator receptor (*uPAR*) [76,77]	• Glycoprotein regulating extracellular matrix degradation and tumour invasion; • Correlated with metastatic potential and poor prognosis; • Shows high lesion detectability with ^68^Ga-uPAR PET in both PCa and gliomas.
